# An 8-week exergame-based exercise training program improves cardiovascular endurance, muscular strength and endurance performances among primary school children in Taiwan

**DOI:** 10.3389/fped.2025.1566838

**Published:** 2025-07-03

**Authors:** Chi-Fang Lin, Po-Fu Lee, Yan-Jhu Su, I-Tung Lin, Hsiao-Fang Kao, Yi-Chuan Hung, Mei-Ling Chao, Chien-Chang Ho

**Affiliations:** ^1^ Department of Physical Education and Sport Sciences, National Taiwan Normal University, Taipei City, Taiwan; ^2^ Office of Physical Education, Fu Jen Catholic University, New Taipei City, Taiwan; ^3^ Department of Sports Management, Minghsin University of Science and Technology, Hsinchu County, Taiwan; ^4^ Department of Leisure Industry and Health Promotion, National Ilan University, Yilan County, Taiwan; ^5^ Department of Gerontology, University of Massachusetts Boston, Boston, MA, United States; ^6^ College of General Education, Chihlee University of Technology, New Taipei City, Taiwan; ^7^ Department of Sport Management, National Taiwan University of Sport, Taichung City, Taiwan; ^8^ Sports Administration, Ministry of Education, Taipei City, Taiwan; ^9^ Department of Nursing, Mei-Ho University, Pingtung County, Taiwan; ^10^ Department of Physical Education, Fu Jen Catholic University, New Taipei City, Taiwan; ^11^ Sports Medicine Center, Fu Jen Catholic Hospital, New Taipei City, Taiwan

**Keywords:** exergaming, physical fitness, school children, Taiwan, physical activity

## Abstract

**Objectives:**

The aim of the present study was to determine the effects of an 8-week exergame-based exercise training program on health-related physical fitness performances among primary school children in Taiwan.

**Materials and methods:**

The study employed a randomized controlled trial (RCT) design, recruiting 68 elementary school children with an average age of 10.76 ± 0.49 years. Participants were randomly divided into an experimental group (*n* = 35) and a control group (*n* = 33). The experimental group completed an 8-week exergame-based exercise training program, performed three times per week. Each session consisted of three one-minute rounds. In contrast, the control group did not undergo any training and continued their usual daily activities. Data collection included demographic surveys, anthropometric assessments, and health-related physical fitness evaluations conducted at baseline (Week 0) and after the intervention (week 8).

**Results:**

After the 8-week exergame-based exercise training program, the results indicated that compared to the control group, the experimental group significantly reduced their 800-m run time [*β* = −21.771, *p* = 0.005 (enhanced cardiovascular endurance)] and increased their performance in bent-leg sit-ups [*β* = 4.036, *p* = 0.021 (muscular strength and endurance)] after adjusting for age, gender, and other health-related physical fitness indicators.

**Conclusion:**

This suggests that 8-week exergame-based exercise training program can be a fun exercise alternative for improving cardiovascular endurance, muscular strength and endurance performances among primary school children in Taiwan.

## 1 Introduction

Regular physical activity plays a crucial role in promoting overall physical and psychological health (1), particularly among children. Engaging in physical activity not only maintain healthy body composition (2) but also contributes to the development of strong muscle strength and endurance (3), enhances cardiovascular fitness (4), and improves academic performance (5, 6) and cognitive function (5, 7).

Eaton et al. reported in a Centers for Disease Control and Prevention (CDC) report that fewer than half of children in the United States meet the current recommendation of at least 60 min of vigorous or moderate-intensity physical activity per day (8). Chang and Wu recruited data concerning school-aged children and adolescents in Taiwan, which including online academic and government databases (9). The findings revealed that the overall physical activity levels of Taiwanese students aged 5–17 years were 20% lower than the standards set by the World Health Organization (WHO). Furthermore, the average exercise duration (including physical education classes) for elementary and junior high school students was only 4.8 h per week. Notably, nearly 40% of students reported engaging in little or no physical activity at all. The reduction in children's physical activity is a complex phenomenon involving multiple factors. Firstly, technological advancements such as smartphones, tablets, and video games have become integral parts of children's daily lives. These devices attract children to spend considerable time in front of screens, significantly reducing their physical activity time (10). Such sedentary behavior not only occupies the time they should spend on physical exercise and outdoor activities but also potentially leads to vision problems (11) and mental health issues, such as anxiety and depression (12–14).

Additionally, academic pressure in modern education systems in Taiwan is a significant factor contributing to the decrease in children's physical activity (9). As academic demands increase, children spend more time on classroom learning and homework, leaving less time for sports and play. The strong emphasis on academic achievement by parents and schools makes it difficult for children to balance study and physical activity (9).

Safety concerns in contemporary society also limit children's outdoor activities. Many parents worry about their children's safety, fearing traffic accidents, crime, or other hazards, thereby restricting their outdoor play. This leads to children spending more time indoors rather than engaging in outdoor sports (15).

The accelerated process of urbanization also impacts children's physical activity. In many urban areas, there is a lack of safe and suitable outdoor spaces and sports facilities for children to play. Moreover, environments dominated by high-rise buildings and heavy traffic are not conducive to outdoor activities for children (16).

Finally, cultural and lifestyle changes have also affected children's physical activity. In the past, outdoor games and sports were essential parts of children's daily lives, but modern lifestyles emphasize indoor entertainment and electronic games (17). This shift makes children more inclined to engage in sedentary activities rather than dynamic ones.

Nevertheless, technology also holds potential to enhance children's physical activity. To address this challenge, exergames that encourage physical activity while maintaining the appeal of digital entertainment have emerged as a promising intervention (18–20). A literature review by Calcaterra et al. suggests that motion-sensing games may be a viable strategy for altering children's obesity and sedentary behavior (21). Additionally, technology may provide opportunities for children to engage in home-based activities in urban areas where outdoor space is limited. Home-based activities may also alleviate parental concerns about the safety of outdoor activities for their children. However, this review contends that motion-sensing games must be grounded in children's self-regulation and the structural integrity of the game itself to yield positive effects (21).

This study targeted children aged 10–12 years due to the developmental transitions inherent in this age group, specifically the increased autonomy in physical activity decision-making, which facilitates better adaptation to exergame-based exercise training (22). Concurrently, this age cohort exhibits a rising prevalence of obesity (23), and physical activity and dietary habits are significantly associated with obesity risk (24).

Hence, exergame-based exercise employed in this study required continuous body movement and dodging of obstacles, including jumping, crouching, squatting, side-stepping, skipping, prone movements, and so on. Past studies of interventions involving moving and various jumping movements (countermovement jump, squat-jump, drop-jump) found that it can improve fitness ability, muscular strength and explosive power (25–27). Therefore, the purpose of this study was to determine the effects of an exergame-based exercise training program on the health-related physical fitness performances among primary school children in Taiwan.

## 2 Materials and methods

### 2.1 Participants

Seventy 5th- and 6th-grade elementary school children were recruited for study participation through school health clinics, physical education curriculum, posters, and emails. Eligibility criteria included the students who did not engage in regular exercise. Students were excluded if they had any medical conditions requiring treatment, neurological, muscular, or skeletal injuries within the last six months, or if they responded affirmatively to any question on the Physical Activity Readiness Questionnaire (PAR-Q). Prior to beginning the study, all participants and their parents or legal guardians were provided with detailed information about the procedures and ethical considerations in accordance with the Declaration of Helsinki. Parental or guardian consent was obtained for all participants. This study was approved by the Institutional Review Board of Fu Jen Catholic University in Taiwan (FJU-IRB C112169).

### 2.2 Experimental design

A randomized controlled experimental design with baseline (pre-test), post-test, and 8-week follow-up was employed in this study. Participants were randomly assigned to either the experimental group (*n* = 35) or the control group (*n* = 35). At the beginning of the study, participants underwent baseline assessments, which included demographic data (grade, age, gender), anthropometric measurements [height, weight, body mass index (BMI)], the PAR-Q, and fitness evaluations related to health. These same measures were reassessed after the 8-week training program. The intervention was delivered by trained physical education teachers within the primary school setting. To minimize external variability, these teachers also conducted weekly phone calls with participants to monitor their physical activity and dietary habits, and to reinforce adherence to consistent daily routines. After that, 2 participants in the control group dropped out of the study due to ill health, and lack of available time. Therefore, 68 participants—35 in the experimental group and 33 in control group—were taken to final evaluation.

### 2.3 Experimental procedure

The experimental group participated in an 8-week school-based exergaming program for exercise training conducted three times per week. The school-based exergaming programs were conducted at the Taoyuan Municipal His Hai Elementary School in Taiwan. The game “Active Arcade” from NEX (NEX Team Inc.) was used for exercise training. A five minutes warm-up and stretching period was carried out to each session. The children then played the exergames for 10 min (3 three-minute rounds with a 30-second rest interval between rounds) followed by a 5-minute cooling down. Each session included three rounds, featuring three one-minute distinct games per round: (1) Laser Dodge: Participants moved to avoid virtual laser beams on the screen. As the difficulty progressed, overlapping beams required dynamic movements such as jumping and squatting. (2) Cone Knockout: This game required participants to move sideways to touch virtual cones displayed on the screen, emphasizing side-stepping movements. (3) Box Attack: Participants positioned themselves boxes that appeared randomly on the screen. New box would only appear after participants successfully entered the previous one, incorporating movements like jumping, squatting, and lying prone. Before beginning the exergaming program, the three games were given as much time as needed to familiarize themselves with the games. These games were chosen for this study because of ease of usage and accessibility (28). In addition, the exercise intensity was monitored with a heart rate (HR) monitor (Polar, Inc., USA) during exergaming program for exercise training. The HR was monitored above 130 beats per minute. The control group did not participate in any structured training and maintained their usual daily activities throughout the study period.

### 2.4 Health-related physical fitness assessments

Five key health-related physical fitness indicators were measured according to the guidelines outlined on the official website of the Taiwan Ministry of Education (17): BMI, muscle strength and endurance (bent-leg sit-ups), explosive power (standing long jump), flexibility (sit-and-reach), and cardiorespiratory endurance (800-m run).

#### 2.4.1 Body composition

Assessed using BMI, calculated as weight (kg) divided by body height squared (m^2^). Body height and weight were recorded without shoes to the nearest 0.1 cm and 0.1 kg using a combined wall-mounted stadiometer and metric balance scale (HW-3070), respectively.

#### 2.4.2 Muscle strength and endurance

Measured using bent-leg sit-ups. Participants performed as many sit-ups as possible within one minute while lying on a mat with knees bent, arms crossed on the chest, and ankles held by a technician. A technician held the participant's ankles firmly for support and conducted the count. The participant's elbows had to touch the corresponding knee (i.e., left touch left, etc.). After each upward movement, the shoulders returned to touch the mat, but touching the mat with the head was not required.

#### 2.4.3 Explosive power

Evaluated through the standing long jump. Participants jumped forward from a starting line using both feet, with the furthest of two attempts recorded in centimeters. They were free to use their arms and legs for countermovement.

#### 2.4.4 Flexibility

Measured with the sit-and-reach test. Participants extended their arms forward while sitting with legs straight, and the furthest point reached on a ruler was recorded. The device used had a measuring scale positioned such that reaching the feet with the fingertips recorded a distance of 25 cm. Before the test, shoes were removed and participants were instructed to slowly reach forward as far as possible with their knees fully extended. The longer distance from two attempts was recorded in centimeters.

#### 2.4.5 Cardiorespiratory endurance

Assessed using an 800-meter run for primary school children. Participants ran in small groups, aiming to complete the distance as quickly as possible after a proper warm-up. The participants ran in groups of six to eight from behind a starting line, and were instructed to try to keep a steady speed, finishing the run as quickly as possible.

All measurements were conducted by trained physical education teachers and nurses. Inspectors from the Ministry of Education ensured adherence to standardized procedures.

### 2.5 Statistical analysis

Data were analyzed using SPSS version 25.0. Independent sample *t*-tests were applied to compare the baseline demographic data, anthropometric measurements, and health-related physical fitness indicators between groups. Paired sample *t*-tests and independent sample *t*-tests were used to evaluate changes in health-related physical fitness indicators pre- and post-training in the experimental and control groups. Additionally, multiple regression analysis was conducted to examine the relationship between pre- and post-test changes across both groups. Results were expressed as mean ± standard error (SE), with statistical significance set at *p* < .05.

## 3 Results

Table 1 presents the descriptive results of all participants, randomly assigned into the experimental group (*n* = 35) and the control group (*n* = 33) after data collection on basic information, anthropometric measurements, and health-related physical fitness assessments. The results revealed no significant differences between the two groups in terms of age, height, weight, body mass index, systolic blood pressure, diastolic blood pressure, 800-meter run, bent-leg sit-ups, sit-and-reach test, and standing long jump as continuous variables (*p* > .05). However, the experimental group had a significantly lower resting heart rate compared to the control group (84.14 ± 1.61 vs. 90.15 ± 1.80 bpm, *p* = 0.015). Additionally, the chi-square test indicated that there were no significant differences between the two groups in terms of the categorical variable of gender (*p* > .05).

**Table 1:** Baseline demographic characteristics of participants.

Variables	Intervention group	Control group	*p*-value
Number	35	33	
Age (years)	10.77 ± 0.08	10.76 ± 0.09	0.909
Gender (male %)	48.6	48.5	0.094
Height (cm)	146.70 ± 1.61	145.86 ± 1.63	0.715
Body weight (kg)	40.65 ± 2.22	44.16 ± 2.19	0.265
BMI (kg/m^2^)	18.54 ± 0.71	20.61 ± 0.89	0.071
HR (bpm)	84.14 ± 1.61	90.15 ± 1.80	**0**.015*
SBP (mmHg)	109.40 ± 2.02	113.15 ± 1.91	0.183
DBP (mmHg)	63.71 ± 1.62	64.58 ± 1.20	0.673
800-m run (seconds)	278.74 ± 10.59	275.42 ± 8.78	0.811
Bent-leg sit-ups (repetitions/min)	28.23 ± 1.60	26.79 ± 1.49	0.513
Sit-and-reach test (cm)	31.34 ± 1.42	30.12 ± 1.41	0.544
Standing long jump (cm)	141.69 ± 5.05	141.36 ± 5.37	0.965

* *p* < 0.05.

Values are expressed as means ± SE.

BF, body fat percentage; BMI, body mass index; DBP, diastolic blood pressure; HR, heart rate; SBP, systolic blood pressure; SE, standard error.

Table 2 presents the results after the 8-week exergame-based exercise training program. In the experimental group, participants showed significant improvements, with the 800-meter run time significantly reduced from pre-test to post-test (278.74 ± 10.59 vs. 250.57 ± 7.86 s, *p* < 0.0001). Additionally, there were significant increases in the number of bent-leg sit-ups (28.23 ± 1.60 vs. 31.86 ± 1.66 repetitions/min, *p* = 0.001), standing long jump distance (141.69 ± 5.05 vs. 151.71 ± 4.93 cm, *p* < 0.0001), and BMI (18.54 ± 0.71 vs. 18.85 ± 0.69 kg/m^2^, *p* = 0.003) from pre-test to post-test. In contrast, the control group showed significant improvements in flexibility, as indicated by higher scores in the sit-and-reach test. (30.12 ± 1.41 vs. 31.52 ± 1.34 cm, *p* = 0.002). However, they also exhibited a significantly higher BMI at post-test compared to pre-test (20.61 ± 0.89 vs. 20.85 ± 0.87 kg/m^2^, *p* = 0.007), which may reflect an increase in body fat rather than muscle mass.

**Table 2:** The comparison of health-related physical fitness measurements between baseline and week 8.

Variables	Intervention group			Control group		
	Baseline	Week 8	*p*-value	Baseline	Week 8	*p*-value
800-m run (seconds)	278.74 ± 10.59	250.57 ± 7.86	<0.001***	275.42 ± 8.78	272.97 ± 9.17	0.602
Bent-leg sit-ups (repetitions/min)	28.23 ± 1.60	31.86 ± 1.66	0.001**	26.79 ± 1.49	26.58 ± 1.11	0.853
Sit-and-reach test (cm)	31.34 ± 1.42	32.37 ± 1.44	0.101	30.12 ± 1.41	31.52 ± 1.34	0.002**
Standing long jump (cm)	141.69 ± 5.05	151.71 ± 4.93	<0.001***	141.36 ± 5.37	145.30 ± 5.13	0.076
BMI (kg/m^2^)	18.54 ± 0.71	18.85 ± 0.69	0.003**	20.61 ± 0.89	20.85 ± 0.87	0.007**

Values are expressed as means ± SE.

BF, body fat percentage; BMI, body mass index; SE, standard error.

*p < 0.05.

**p < 0.01.

***p < 0.001.

Table 3 demonstrates that the experimental group exhibited significantly greater improvements in the 800-meter run time (−28.17 ± 5.32 vs. −2.45 ± 4.66 s, *p* = 0.001), bent-leg sit-ups (3.63 ± 1.05 vs. −0.21 ± 1.14 repetitions/min, *p* = 0.015), and standing long jump distance (10.03 ± 2.02 vs. 3.94 ± 2.15 cm, *p* = 0.043) compared to the control group.

**Table 3:** Changes in health-related physical fitness measurements after an 8-week exergame-based exercise training program.

Variables	Intervention group	Control group	*p*-value
800-m run (seconds)	−28.17 ± 5.32	−2.45 ± 4.66	0.001**
Bent-leg sit-ups (repetitions/min)	3.63 ± 1.05	−0.21 ± 1.14	0.015*
Sit-and-reach test (cm)	1.03 ± 0.61	1.39 ± 0.41	0.626
Standing long jump (cm)	10.03 ± 2.02	3.94 ± 2.15	0.043*
BMI (kg/m^2^)	0.31 ± 0.10	0.24 ± 0.08	0.577

Values are expressed as means ± SE.

BF, body fat percentage; BMI, body mass index; SE, standard error.

*p < 0.05.

**p < 0.01.

***p < 0.001.

Table 4 presents the results of multiple regression analysis examining the relationship between the changes in health-related physical fitness indicators before and after the intervention in both the experimental and control groups. Using the control group as the baseline and adjusting for confounding factors such as age and gender, the experimental group demonstrated a significant reduction in 800-meter run time [*β* = −25.857, *p* = 0.001 (improved cardiovascular endurance)], an increase in bent-leg sit-ups [*β* = 3.836, *p* = 0.014 (improved muscular strength and endurance)], and an improvement in standing long jump performance [*β* = 6.094, *p* = 0.045 (improved explosive power)]. Furthermore, after adjusting for age, gender, and other health-related physical fitness indicators, the experimental group still showed a significant reduction in 800-meter run time [*β* = −21.771, *p* = 0.005 (improved cardiovascular endurance)] and an increase in bent-leg sit-ups [*β* = 4.036, *p* = 0.021 (improved muscular strength and endurance)] compared to the control group.

**Table 4:** Multiple linear-regression analysis of changes in health-related physical fitness measurements with the control group as comparator while adjusting for potential confounders.

Variables	Group	Model 1 (Age-and gender adjusted)			Model 2 (adjusted^a^)		
		*β*	SE	*p*-value	*β*	SE	*p*-value
800-m run (seconds)	Intervention group	−25.857	7.103	**0.001** **	−21.771	7.511	0.005**
	Control group	Reference	—	—	Reference	—	—
Bent-leg sit-ups (repetitions/min)	Intervention group	3.836	1.512	0.014*	4.036	1.709	0.021*
	Control group	Reference	—	—	Reference	—	—
Sit-and-reach test (cm)	Intervention group	−0.368	0.754	0.627	−1.200	0.861	0.169
	Control group	Reference	—	—	Reference	—	—
Standing long jump (cm)	Intervention group	6.094	2.980	0.045*	3.819	3.408	0.267
	Control group	Reference	—	—	Reference	—	—
BMI (kg/m^2^)	Intervention group	0.072	0.131	0.582	0.004	0.152	0.979
	Control group	Reference	—	—	Reference	—	—

^a^ Adjusted for age, gender, and change in other health-related physical fitness measurements.

BF, body fat percentage; BMI, body mass index; SE, standard error; *β*, regression coefficient.

*p < 0.05.

**p < 0.01.

***p < 0.001.

## 4 Discussion

This study aims to investigate the effects of 8-week exergame-based exercise training program on the health-related physical fitness of Taiwanese primary school children. The main finding of this trial was that participating in thrice-weekly sessions for eight weeks improved cardiovascular endurance (as measured by the 800-meter run) and muscular strength and endurance (as measured by bent-leg sit-ups) compared to a control group that received no intervention.

We found no significant differences between the experimental and control groups in terms of baseline characteristics and health indicators, including age, height, weight, body mass index, systolic and diastolic blood pressure, and measures such as the 800-meter run, bent-leg sit-ups, sit-and-reach test, and standing long jump. However, the experimental group had a significantly lower resting heart rate than the control group, suggesting that the experimental group already had better cardiorespiratory function prior to training.

Additionally, after eight weeks of exergame training, we observed significant improvements in multiple health-related physical fitness indicators among the children in the experimental group. Notably, the experimental group showed considerably better results in the 800-meter run, bent-leg sit-ups, standing long jump, and BMI. In contrast, the control group also experienced improvements in certain indicators, such as the sit-and-reach test and BMI, though these improvements were less pronounced than those in the experimental group. We implied this may be because the control group subjects experienced some improvements in certain indicators due to their greater familiarity with the testing procedures and content during the follow-up tests compared to the initial tests. Our study, similar to some previous research findings, indicates that regular physical activity can enhance the fitness performance of school children (29). Additionally, the BMI of primary school children can change due to various factors, such as ongoing height development, making it less stable compared to adults (30).

Notably, the significant improvement in the 800-meter run, bent-leg sit-ups, and standing long jump tests was significantly greater in the experimental group than in the control group. This further substantiates the positive effects of exergame training on health-related physical fitness. It is possible that exergames are considered to be light to moderate physical activity (31–33), which can lead to increased fitness performance. Past finding had been found that energy expenditure increases by an average of 222% and heart rate increases by 64%, while energy expenditure is significantly higher in games involving the lower extremities (31).

The results of multiple regression analysis showed that, even after controlling for confounding factors such as age and gender, the improvements in various indicators were still significant in the experimental group compared to the control group. This suggests that exergame training provides stable and reliable improvements in health-related physical fitness indicators such as cardiorespiratory endurance, muscular strength and endurance, and explosive power. Our study results are similar to those of previous studies, showing that the physical fitness performance of children in the experimental group with regular physical activity was superior to that of the children in the control group (29). Moreover, Ketelhut et al. conducted a three-month intervention in which the experimental group participated in additional exergame training twice weekly, for 20 min per session (34). Although the physical fitness tests assessed in their study differed from those employed in the present study, their findings similarly demonstrated significant improvements in explosive power, speed, and cardiorespiratory endurance among elementary school students following exergame participation.

In summary, the results of this study strongly support the effectiveness of exergame training in improving health-related physical fitness. These findings offer valuable references for exercise training and health promotion and lay the groundwork for future research. Exergame training's positive impact on health-related physical fitness provides a novel approach to exercise training. Compared to traditional fitness training, exergame training is more engaging and diverse, attracting and retaining more participants. This is significant in encouraging exercise and improving health status, particularly for individuals who may not be interested in or able to maintain traditional training methods.

Additionally, the study's results provide empirical support for formulating exercise prescriptions and health management plans. Exergame training can be tailored to target improvements in various health indicators such as cardiorespiratory function, muscle strength, and flexibility, effectively helping individuals enhance their overall health. This training approach may be particularly beneficial for 10–12-year-old children, who require specific exercise prescriptions. However, if future research aims to target younger or older children, adjustments to exergame-based exercise design and training intensity will be necessary. For younger children, simplification of game content is required, whereas for older children, increased challenge should be incorporated, which will maybe lead to potentially resulting in similarly from the current study.

However, despite the positive effects of exergame training on health-related physical fitness shown in this study, there are still potential limitations and areas that require further investigation. For example, the sample size of this study is relatively small, and the training duration is short, so long-term effects and responses from different populations still need to be further observed. Additionally, the specific design, suitable populations, and optimal implementation methods of exergame training require more in-depth research and discussion.

## 5 Conclusion

Overall, this study unequivocally demonstrate that an 8-week exergame-based training program effectively enhances cardiovascular endurance, muscular strength, muscular endurance, and explosive power in Taiwanese primary school children. Compared to the control group, the experimental group exhibited significant improvements across multiple health-related physical fitness indicators. This study underscores the potential of exergames as an engaging and efficacious exercise modality for improving physical fitness in this population. Future research should further explore the long-term effects of exergame training and extend the investigation to diverse populations, such as older adults, adolescents, and individuals with specific health conditions, to validate its applicability. Additionally, in-depth investigations into the physiological mechanisms and motivational factors underlying the observed improvements in physical fitness, as well as the incorporation of qualitative research to understand participants' experiences, are crucial directions for future studies. Furthermore, the potential clinical applications of exergames, such as in cardiac or musculoskeletal rehabilitation, warrant further exploration.

## Data Availability

The raw data supporting the conclusions of this article will be made available by the authors, without undue reservation.
